# Extraction and Synthesis of Typical Carotenoids: Lycopene, β-Carotene, and Astaxanthin

**DOI:** 10.3390/molecules29194549

**Published:** 2024-09-25

**Authors:** Yuxuan Jiang, Jingyi Ye, Yadong Hu, Jian Zhang, Wenhui Li, Xinghu Zhou, Mingzhou Yu, Yiyang Yu, Jingwei Yang, Wenge Yang, Jinchi Jiang, Jie Cui, Yonghong Hu

**Affiliations:** 1 College of Biotechnology and Pharmaceutical Engineering, Nanjing Tech University, Nanjing 211816, China; jyxxxx@njtech.edu.cn; 2 College of Food Science and Light Industry, Nanjing Tech University, Nanjing 211816, China; 202361119019@njtech.edu.cn (J.Y.); four-leaf-clover@njtech.edu.cn (J.Z.); lwhnjtec@163.com (W.L.); ymz@njtech.edu.cn (M.Y.); yuyy@njtech.edu.cn (Y.Y.); jiangjinchi@126.com (J.J.); 3 Jiangsu Innovation Center of Marine Bioresource, Jiangsu Coast Development Investment Co., Ltd., Nanjing 210019, China; huyadong@jsyhkf.com (Y.H.); zhouxinghu@jsyhkf.com (X.Z.); 4 Key Laboratory of Coastal Salt Marsh Ecosystems and Resources, Ministry of Natural Resources, Nanjing 210006, China; sxy03280203@126.com; 5 School of Pharmaceutical Sciences, Nanjing Tech University, Nanjing 211816, China; yangwenge11@163.com

**Keywords:** carotenoids, lycopene, β-carotene, astaxanthin, biosynthesis, extraction

## Abstract

Carotenoids are tetraterpene compounds acting as precursors to vitamin A, with functions that include protecting eyesight, enhancing immunity, promoting cell growth and differentiation, and providing antioxidative benefits. Lycopene, β-carotene, and astaxanthin are particularly critical for health and have diverse applications in food, health products, and medicine. However, natural carotenoids are encased within cell structures, necessitating mechanical methods to disrupt the cell wall for their extraction and purification—a process often influenced by environmental conditions. Thus, improving the efficiency of carotenoid extraction from natural resources is of great interest. This review delves into the research progress made on the extraction processes, structures, and biological functions of carotenoids, focusing on lycopene, β-carotene, and astaxanthin. Traditional extraction methods primarily involve organic solvent-assisted mechanical crushing. With deeper research and technological advancements, more environmentally friendly solvents, advanced machinery, and suitable methods are being employed to enhance the extraction and purification of carotenoids. These improvements have significantly increased extraction efficiency, reduced preparation time, and lowered production costs, laying the groundwork for new carotenoid product developments.

## 1. Introduction

Carotenoids, a class of natural pigments found in plants, impart vivid colors like orange, yellow, or red to some fruits and vegetables. They possess a wide range of physiological functions beneficial to humans, including anticancer [1] and antioxidative properties [2], the promotion of intestinal microecology [3], the alleviation of inflammatory respiratory disease symptoms [4], and a reduction in glaucoma risk [5]. These functions exhibit high bioavailability and research value. Consequently, carotenoid acquisition has become a prominent research topic. Due to safety concerns over chemically synthesized carotenoids and a preference for natural products, the focus has shifted to two main sources: natural extracts and biosynthesis [6]. While biosynthesis presents industrial production challenges due to low yields, natural extraction sources are diverse, including plants, algae, microorganisms, and food processing waste.

To date, over 700 carotenoids have been identified in nature, with lycopene, β-carotene, and astaxanthin being the most extensively studied due to their biological functions. Carotenoids are categorized into two groups based on their chemical structure: (1) carotenes such as lycopene, β-carotene, and α-carotene consist solely of carbon and hydrogen. (2) Xanthophylls, like zeaxanthin, meso-zeaxanthin, and lutein, serve as macular pigment carotenoids [7].

**Figure 1:**
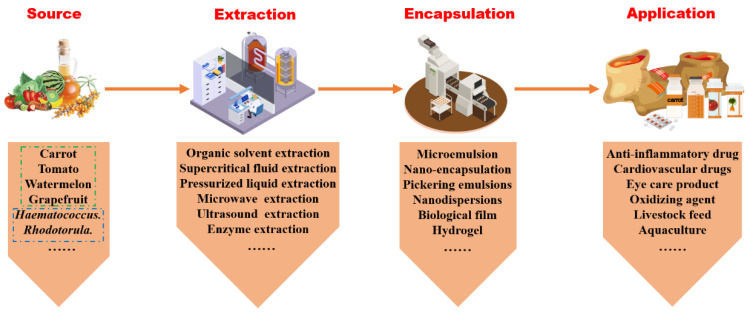
The process of carotenoids from source to product.

The molecular structure of carotenoids is highly sensitive, and it is particularly prone to photodegradation and breakdown under light exposure (especially ultraviolet (UV) exposure), high temperatures, and the presence of oxygen. Therefore, carotenoids are easily degraded when exposed to light, heat, and oxidizing agents. Careful handling is essential to prevent structural alterations that could render them inactive or unstable. The yield and purity of extracted carotenoids can differ based on the chosen extraction and purification techniques and the solvents used, as illustrated in Figure 1.

Most carotenoids reside within cellular compartments. Conventional organic solvent extraction techniques and green solvents are solvents that are environmentally friendly during production and processing and are non-toxic, biodegradable, and usually non-flammable. These solvents have all struggled to efficiently penetrate the intracellular environment to directly contact carotenoids. Hence, physical methods such as microwave and ultrasonication are necessary to enhance the actual yield efficiency of carotenoid collection. The choice of extraction method and organic solvents impacts not only the efficiency of carotenoid extraction but also their biological activity. Post-extraction and purification, various packaging methods, including embedding, nano-encapsulation [8], microemulsion preparation [9], and edible membrane production [10], are employed to preserve the stability and bioactivity of carotenoids.

The burgeoning consumer preference for natural ingredients, coupled with the enhanced understanding of the health benefits conferred by carotenoids, is collectively propelling the market demand for functional carotenoid products. This demand is anticipated to reach a value of USD 1.45 billion in 2024. The global carotenoid market is forecast to expand at a compound annual growth rate (CAGR) of 5.4%, culminating in a market value of USD 2.45 billion by the close of 2034 [11]. Consequently, technological innovations in extraction processes and the formulation techniques are essential to cater to the expanding carotenoid market. The aim of this paper is to review the properties and roles of three specific carotenoids, lycopene, β-carotene, and astaxanthin, and summarize domestic and international research on carotenoid extraction methods. This will provide theoretical references for the extraction and purification of carotenoids.

## 2. Physicochemical Characteristics of Tetraterpenoids

**Figure 2:**
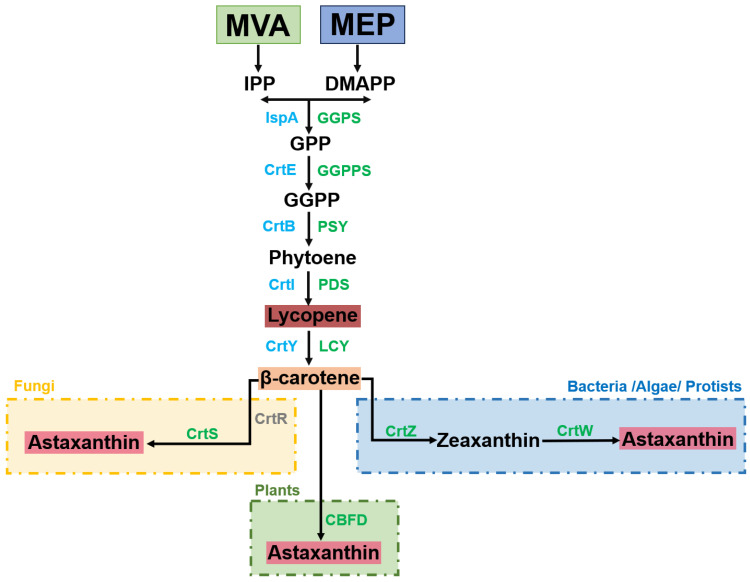
Carotenoid biosynthetic pathway. Enzymes from IPP and DMAPP to β-carotene are shown in blue for bacteria and green for plants/algae as follows: the mevalonate pathway (MVA), the 2-C-methyl-d-erythritol-4-phosphate pathway (MEP), isopentenyl diphosphate (IPP), dimethylallyl diphosphate (DMAPP), farnesyl pyrophosphate synthase (IspA), geranyl pyrophosphate (GPP), geranylgeranyl diphosphate (GGPP), geranyl pyrophosphate synthase (GGPS), geranylgeranyl diphosphate synthase (CrtE/GGPPS), phytoene synthase (CrtB/PSY), phytoene desaturase (CrtI/PDS), lycopene-cyclase (CrtY/LCY), astaxanthin synthase (CrtS), the auxiliary cytochrome P450 reductase (CrtR), β-carotenoids ketoacidase (CrtW), β- Carotene hydroxylase (CrtZ), and carotenoids β-ring 4-dehydrogenase (CBFD).

Lycopene is the initial tetraterpenoid in the carotenoid metabolic pathway [12]. It is synthesized from geranylgeranyl diphosphate (GGPP), which is produced in plants, fungi, and bacteria through the mevalonate pathway (MVA) and the 2-C-methyl-d-erythritol-4-phosphate (MEP) pathway. Figure 2 illustrates the conversion of GGPP into lycopene through a series of synthesis steps and dehydrogenation transitions involving IspA (FPP synthase), phytoene synthase, and phytoene desaturase [13]. Lycopene is subsequently converted into β-carotene by lycopene cyclase [12,14]. In fungi, β-carotene synthesis relies on key enzyme astaxanthin synthase (CrtS) and cytochrome P450 reductase (CrtR), which are both members of the cytochrome P450 protease family. Some bacteria, algae and protozoa, can further synthesize astaxanthin through β-carotene ketoacidase (CrtW) and β-carotene hydroxylase (CrtZ), whereas plants require carotenoid β-ring 4-dehydrogenase (CBFD) for this conversion [15,16,17].

### 2.1. Lycopene

Lycopene is a tetraterpenoid pigment and a needle crystal [18] found in various plants, including tomatoes [19], citrus fruits [20], watermelons [21], carrots [22], and grapefruits [23]. It is insoluble in water but soluble in chloroform and benzene oil. Its molecular weight is 536.85 u(unit), and its chemical formula is C_40_H_56_. The molecular structure of lycopene comprises a straight-chain hydrocarbon with 11 conjugated double bonds and 2 non-conjugated double bonds, as shown in Table 1. Thus, lycopene is highly sensitive to light, and exposure can significantly accelerate its degradation. Additionally, lycopene is unstable at high temperatures and is prone to oxidation and isomerization. While it is relatively stable under aerobic conditions, oxidation reactions may occur in high-oxygen environments.

In 1983, researchers demonstrated that lycopene has excellent antioxidant properties and free radical scavenging capabilities [24,52]. Subsequent research has revealed that lycopene also possesses anti-inflammatory [25], anticancer [26], and cardiovascular disease prevention effects [27], making it a valuable resource in medicine, biology, animal husbandry, and other fields. Leh et al. found that, due to the antioxidant effects of lycopene, taking lycopene supplements reduced oxidative stress in the pancreatic β-cells, which are responsible for the production of insulin, resulting in a 37% increase in insulin levels in diabetic patients [29]. Furthermore, oxidative stress was reduced in patients with type II diabetes. Lycopene has neuroprotective benefits and has been used successfully to treat Alzheimer’s disease [28]. It has also been shown to suppress the proliferation of gastric cancer cells without affecting normal gastric epithelial cells, making it a promising drug for the targeted therapy of gastric cancer [30].

Lycopene can also serve as a feed supplement in animal production. It has been shown to improve immunity, metabolism, and reproductive function in animals [53]. For example, the inclusion of 200 mg/kg of lycopene in pig feed resulted in a significantly improved muscle redness value (4.10 to 4.58), intramuscular fat content (3.26% to 3.98%), and crude protein content (22.93% to 25.30%) of pork [31].

### 2.2. β-Carotene

β-carotene is an important source of vitamin A for humans [54]. It enhances the absorption of vitamin A and is commonly found in plants, such as carrots and citrus fruits, which gives them an attractive orange or yellow hue. β-carotene is insoluble in water, propylene glycol, glycerol, acids, or alkalis. It is soluble in ether, petroleum ether, cyclohexane, and vegetable oils and is easily soluble in carbon disulfide, benzene, and chloroform [55].

As indicated in Table 1, β-carotene functions as a lipid radical scavenger and singlet oxygen quencher [56]. When contrasted with astaxanthin and lycopene, β-carotenoids demonstrate a notably lower sensitivity to light. Nonetheless, exposure to ultraviolet light can still hasten their degradation. Similar to other carotenoids, β-carotenoids are unstable at high temperatures and are prone to oxidation and isomerization. Under aerobic conditions, β-carotenoids tend to undergo oxidation reactions in high-oxygen environments.

In medicine, research has shown that the moderate supplement of β-carotene can reduce the risk of coronary heart disease [57] and certain types of cancer [58], boost the immune system [33] and prevent age-related macular degeneration [34]. Nimbalkar observed that the continuous treatment of rats with 20 mg/kg of β-carotene for 14 days reduced all indications of type II diabetes mellitus [32]. This research suggests that β-carotene improves glycometabolism and oxidative state in diabetic rats. However, the integration of β-carotene into various food systems is limited due to its low water solubility and sensitivity to breakdown under light, heat, and oxygen conditions [59]. Encapsulation technology has been employed to improve the stability of β-carotene, with various carriers used to encapsulate it and increase its stability. The highest β-carotene encapsulation efficiency achieved was 92% [60], allowing for its use in a wider variety of applications.

### 2.3. Astaxanthin

Astaxanthin exhibits antioxidant, antibacterial, and antiapoptotic properties and is also effective in scavenging free radicals [61]. Astaxanthin can be extracted from animal sources, while other carotenoids are less abundant in animals or humans to be useful for extraction. Unlike lycopene and β-carotene, which are composed solely of carbon and hydrogen, astaxanthin also contains oxygen-containing functional groups [62] (referred to Table 1). Its chemical formula is C_40_H_52_O_4_, and its molecular structure features a conjugated double-bond chain with an unsaturated ketone and a hydroxyl at the end of the chain. These functional groups can attract or donate unpaired electrons to free radicals, scavenging them and exhibiting antioxidant properties [35].

Astaxanthin, like other carotenoids, is particularly sensitive to light, heat, and oxygen. Astaxanthin is insoluble in water but soluble in fats, chloroform, acetone, benzene, and most other organic solvents. In 1949, astaxanthin was isolated in small quantities from marine crustacean shells, signifying its initial discovery [63]. According to Nair’s study, astaxanthin can inhibit NF-κB or JAK/STAT pathways, suppressing pro-inflammatory cytokine production [64]. Hirotaka et al. assessed the superoxide scavenging activity before and after astaxanthin supplementation, observing an increase in superoxide dismutase activity from approximately 18.2 U/mL to 19.9 U/mL over two weeks. Additionally, skin biopsies performed after UV irradiation with collagen hydrolysate supplemented with 2 mg/day of astaxanthin showed a significantly increased expression of type I procollagen in the astaxanthin group compared to the placebo group [36,37]. Liu et al. found that appropriate astaxanthin supplementation (4–12 mg/day) for three consecutive months significantly increased the production of type I procollagen in healthy individuals. Due to these physiological functions of astaxanthin, the appropriate supplementation of astaxanthin in the diet can prevent glaucoma (through antioxidant defense) [65], alleviate symptoms of associated vascular litis [66], and prevent cerebral hemorrhage (by inhibiting reactive oxygen species and activating antioxidant defenses) [67].

Astaxanthin is primarily found in *Haematococcus pluvialis*, *Chlorella*, *Cladophora aegagropila*, and *Phaffia rhodozyma* [68]. It has various applications, including in aquaculture [69] and the synthesis of antimicrobial drugs [70]. However, due to its scarcity and high cost, the extraction and purification of natural astaxanthin can be challenging, which limits its applicability.

### 2.4. Other Important Carotenoids

In addition to well-known carotenoids, such as lycopene, β-carotene, and astaxanthin, there are many other equally important carotenoids, such as lutein, zeaxanthin, and canthaxanthin. These carotenoids are also rich in nutritional value, possessing anti-inflammatory and antioxidant properties. For tabulated data, please refer to Table 1. Lutein and zeaxanthin are the only carotenoids that accumulate in the retina, particularly in the macula and are known as macular pigments. Their antioxidant properties are positively associated with brain and eye development and protection [41,49]. Additionally, lutein and zeaxanthin have neuroprotective properties and may be used to treat neurodegenerative diseases [43]. Canthaxanthin can improve animals’ resistance to hypoxic stress and promote their reproductive development by increasing the secretion of reproductive hormones in hens [46]. It can also protect ram sperm from oxidative stress [44].

However, the market demand for lutein, zeaxanthin, and canthaxanthin is limited compared to that for lycopene, β-carotene, and astaxanthin, which are more commonly used. Furthermore, it is worth noting that lutein, zeaxanthin, and canthaxanthin constitute a lower percentage of the plant or microbial content compared to the other three carotenoids. This is due to the challenges involved in extracting and isolating them, which often results in the extraction of carotenoids in their composite form. Consequently, there have been limited studies on the extraction of lutein, zeaxanthin, and canthaxanthin in isolation.

## 3. Extraction of Carotenoids

The characteristics of carotenoids require more careful consideration of solvent and extraction methods during the process of extraction and separation. This makes extracting significant quantities of carotenoids to fulfill human demand problematic, resulting in significant losses, particularly during the extraction process. Carotenoids are primarily extracted from three sources: plants, fungi, and bacteria, with a small portion derived from animals. Cell wall destruction is required prior to extraction as carotenoids are found largely in plant cytoplasm or within bacterial and fungal cells. The yield of extracted carotenoids has significantly increased over time due to technical developments and improvements in extraction processes.

**Figure 3:**
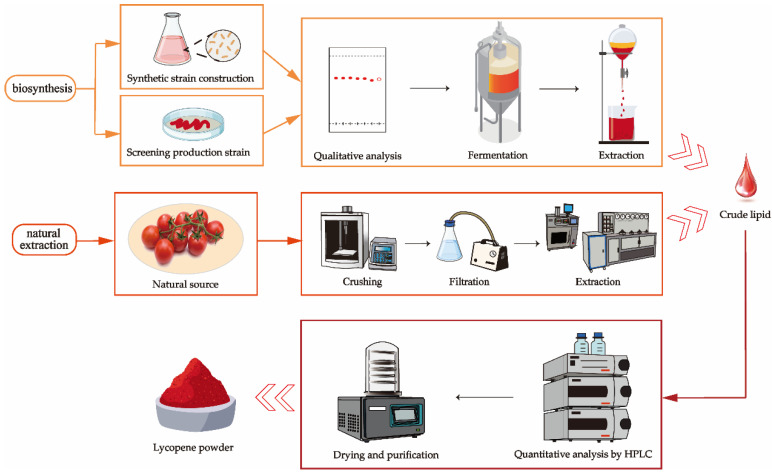
Carotenoid extraction process.

Carotenoid extraction can be divided into two methods: extraction from natural resources and extraction after microbial fermentation or synthesis. The extraction of carotenoids from natural sources like carrots and tomato peels typically involves pretreatment steps, such as removing dirt and impurities, cutting, juicing, pureeing, and filtering, followed by mechanical and chemical extraction methods. The extracted carotenoids are then dried and quantitatively analyzed. Microbial biosynthesis requires the development of engineered strains capable of producing carotenoids or the screening and isolation of naturally carotenoid-producing strains. Following qualitative analysis, these strains are cultured in a fermenter to produce large quantities of carotenoids, which are then extracted using mechanical and chemical methods. The resulting product is then dried and subjected to quantitative analysis, as shown in Figure 3.

In 1966, carotenoids and chlorophyll were extracted, isolated, and quantitatively measured from leaves and algae using thin-layer chromatography [71]. However, chromatography can only extract a limited number of carotenoids, which cannot be used as raw materials for further processing. By 1970, the microbial biosynthesis of carotenoids had been discovered along with extraction and purification from natural sources, but the output was still insufficient to meet manufacturing needs [72].

The core databases, PubMed, Web of Science, and Sciencedirect, were used to search for terms associated with carotenoid extraction, such as ‘carotenoid extraction’, ‘extraction of fat-soluble substances’, and ‘assisted extraction’, among others. The selection criteria included the following: (1) original experimental research articles and related review articles; (2) articles written in English; and (3) papers released between 2013 and 2023. During the screening phase, articles lacking sufficient data or information, such as important methodological details, results, or references, were eliminated. The article’s title and abstract served as the first selection criteria. Potentially appropriate full-text publications were then found and assessed for eventual inclusion in the research.

The methods for extraction can be categorized into several types, including organic solvent extraction, green solvent extraction, microwave-assisted extraction (MAE), ultrasound-assisted extraction (UAE), supercritical fluid extraction (SFE), pressurized liquid-assisted extraction (PLE), pulsed electric field-assisted extraction (PEF), and enzyme-assisted extraction (EAE).

**Figure 4:**
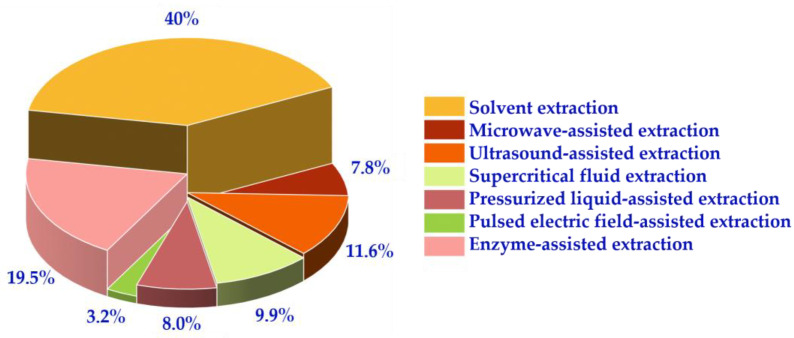
The proportion of various extraction methods in the core database.

Figure 4 presents an analysis of the research articles searched, revealing that 40% of the literature used the solvent method for extracting carotenoids. The remaining 41% employed other assisted extractions such as EAE, SFE, and UAE. These four extraction methods used show promise for the development and use of carotenoid extraction. The processes require further investigation and improvement to increase extraction efficiency and reduce costs. The aim of this work is to provide a comprehensive overview of the research methodology and findings related to the industrial application of carotenoids.

### 3.1. Solvent Extraction

One of the most common and traditional extraction methods is organic solvent extraction. This method involves dissolving carotenoids in organic solvents such as ethyl acetate, methanol, or ethanol. There are two general categories for the extraction process: solid–liquid extraction and liquid–liquid extraction. The nature of the extract determines which extraction agent is optimal for organic solvent extraction. Selecting a solvent with low solubility for contaminants and high solubility for carotenoids is crucial. This can be accomplished through methods such as Soxhlet extraction, crushing, and shock extraction. Additionally, liquid–liquid extraction can be employed to separate specific components from a solution by utilizing their distinct partition coefficients in two immiscible reagents, allowing them to transfer from one solvent phase to another and separate from other ingredients. Compared to solid–liquid extraction, liquid–liquid extraction is simpler to operate and facilitates automated high-throughput procedures. However, its efficacy is lower, necessitating multiple extractions to obtain highly pure compounds. In contrast, efficient solid–liquid extractions require specialized equipment, leading to higher costs and more complex procedural stages.

Table 2 indicates that Poojary et al. successfully recovered lycopene from tomato waste with a high purity level (98.3%) and recovery rate (94.7%) using a 1:3 (*v*/*v*) n-hexane/acetone concentration [19]. Additionally, carotenoids were extracted from the pericarp of wood betel seed using ethyl acetate, yielding 271 mg/100 g (dry weight) after 150 min [73].

However, traditional organic solvent extraction presents several drawbacks, including lengthy extraction times, low efficiency, and environmental concerns. In 2012, Chemat et al. introduced the concept of ‘green extraction of natural products’, which involves the use of alternative solvents and renewable resources, along with six principles for the extraction of natural products. Since then, there has been an increasing focus on developing alternative, ecologically friendly solvents to replace conventional chemical solvents in the separation and extraction of bioactive components. Water, ionic liquids, deep eutectic solvents (DESs), and supercritical CO_2_ have become important members of the green solvent family [103]. Compared to traditional organic solvents, DESs represent a new class of green solvents with advantages such as easy production, high biocompatibility, excellent biodegradability, efficient solvation capability, strong thermal stability, and low volatility [104]. After blending eucalyptus oil with menthol for 20 min, the yield of extracted carotenoids reached approximately 359.3 mg/100 g (fresh weight) [74]. Ghany et al. fermented the exoskeletons of Penaeus japonicus and Penaeus semisulcatus with Saccharomyces cerevisiae, obtaining astaxanthin at a concentration of 8.5 mg/g after extraction with a 1:1 (*v*/*v*) hexane/acetone mixture [75].

The oleaginous yeast *Rhodosporidium toruloides* can convert low-cost carbon sources like cornstarch and tapioca starch into high-value carotenoids, which have diverse applications across industries, including medicine, food, livestock, and agriculture. However, *R. toruloides* also produces a significant amount of lipids during carotenoid synthesis, which increases the complexity and cost of isolating the metabolites. Most research focuses on two aspects as follows: enhancing the proportion of carotenoids in microbial metabolites through biosynthesis and improving extraction procedures to efficiently separate carotenoids from mixed metabolites. Liu et al. achieved a recovery rate of 78.7% for high-purity carotenoids from *R. toruloides* after saponification with an aqueous KOH solution, extraction with alcohol and hexane, thereby reducing the cost of extraction from oleaginous yeast [76].

FAEEs (fatty acid ethyl esters) also serves as green organic solvents that can be applied in carotenoid extraction. The FAEE method and ultrasonic-assisted extraction were employed to extract β-carotene and lycopene from tomato waste. The FAEE method alone achieved a β-carotene extraction concentration of 38.3 mg/100 g/15 min, which increased to 49.7 mg/100 g/15 min with the combined use of FAEE and UAE. For lycopene, the maximum concentration of 101.4 mg/100 g was reached in just 6 min using a combination of FAEE and UAE, compared to 15 min with FAEE alone [77]. Research has demonstrated that supramolecular solvents (SUPRAS) can extract up to 1 mg/g (dry weight) of total carotenoids from *Scenedesmus* sp. within five minutes [78]. In comparison to the traditional organic solvent extraction presented in Table 2, green solvents like SUPRAS and FAEE offer certain advantages in terms of extraction efficiency and speed.

Enhancing the recovery rate of solvent extraction and reusing the solvents can significantly reduce the environmental pollution caused by organic solvents while also decreasing the cost of extraction. The recovery of solvents can be achieved through separation technologies or by employing renewable solvents. These include ethanolic carboxylic acids mixtures and ethyl acetate [105] and the use of eutectic solvents such as menthol [106], ethyl acetate [107], ethyl lactate [108], and norflurane [109], which can also facilitate the recycling of solvents post-extraction. 

Furthermore, due to the predominant cellular localization of carotenoids, extraction using only organic solvents proves inefficient. Therefore, various physical methods are being employed as adjuncts to enhance extraction efficiency.

### 3.2. Ultrasound-Assisted Extraction

Ultrasound enhances extraction by generating mechanical vibrations that produce strong shear forces and convert internal friction into heat. This raises temperatures, reducing the viscosity of incompressible fluids or increasing that of compressible ones, thereby rupturing plant cell walls and improving their dispersion in the solvent. This increases the contact area and mass transfer efficiency between the solvent and the target substance. Compared to other extraction procedures, ultrasonic extraction does not harm the structure or activity of carotenoids. It is compatible with most organic solvents, regardless of the polarity of the solution composition or molecular weight. This property can significantly reduce the extraction time of carotenoids, preserve their biological activity, and improve product quality [110]. Furthermore, research into the extraction of other antioxidant substances has revealed that UAE can significantly reduce the impact on the bioactivity of extracted compounds, owing to its short operational time and lower working temperature [111].

Table 2 shows that Luengo et al. used solvent-assisted pressure UAE with a hexane/ethanol mixture to increase the extraction of carotenoids from tomato waste. They were able to reduce the quantity of hexane to 25% without affecting the extraction rate [79]. Goula et al. employed ultrasonic extraction to extract carotenoids from pomegranate wastes and achieved a recovery rate of 93.8% (0.3255 mg of carotenoids/100 g of dry peels) [80]. Under the condition of 2:1 (*v*:*v*) ethanol/hexane, the maximum carotenoid extracted from Chlorella vulgaris and Microalgae *Purpuricus porphyrinaceus* was 6435.60 µg/g by UAE [81]. Gu et al. extracted carotenoids from *Rhodobacter sphaeroides* by UAE and achieved approximately 664 µg/g for 40 min [112]. UAE, combined with menthol and camphor (a combination of DES), increased the carotenoid extraction rate from 163.5 mg/100 g (fresh weight) to 653.5 mg/100 g (fresh weight) compared with DES alone [82]. Research by Elsa et al. found that the yield and purity of carotenoids extracted from Sechium edule using UAE were higher than those obtained through maceration and MAE [113]. Hemanta et al. optimized UAE for passion fruit peel using olive oil as a solvent, achieving an extraction rate of 91.4% compared to 86.9% with MAE under the same conditions, and when comparing the energy density of both methods, UAE proved superior [114]. The choice of solvent and the solvent-to-solid ratio in UAE were also significant factors affecting extraction efficiency and bioactivity [115]. H. Hadiyanto maximized the yield of β-carotene to 1.38 µg/mL by adjusting the solvent-to-solid ratio to 1:6 [116].

Researchers have started investigating the use of UAE for the direct extraction of carotenoids or incorporating a single organic solvent to mitigate the environmental impact and potential hazards associated with residual organic solvents under the framework of green environmental protection. They used response surface methods to optimize extraction conditions with 51% ethanol and recovered a total of 31.82 µg/g of carotenoids from carrot waste residue for 17 min at 32 °C. The study found that the sample contained 14.89 µg/g of β-carotene, 5.77 µg/g of lutein, and 2.65 µg/g of lycopene [83]. The researchers used ultrasound to break the cell structure of *Rhodotorula glutinis* in an aqueous medium, resulting in the formation of small droplets of carotenoids coated with phospholipid, creating a uniform microemulsion with an average particle size of 230 nm. The carotenoid recovery rate of *R. glutinis* was 82%, and the carotenoid content obtained was approximately 25 mg/L [84]. The use of UAE and MAE technology enabled the extraction of carotenoids (26.91–34.35 mg/100 g) from *Hippophae rhamnoides* (Sea buckthorn) pomace, using edible oils (corn and olive oils) as a green solvent instead of organic solvents [85].

### 3.3. Supercritical Fluid Extraction

The SFE method utilizes the distinctive characteristics of supercritical fluid to extract carotenoids. Supercritical fluid exhibits properties of both a gas and a liquid and in its supercritical state, its density and solving properties increase, enabling the efficient extraction of carotenoids. Other co-solvents can be added to further enhance the extraction process. Many critical fluids, such as carbon dioxide, ammonia, and water, are non-toxic and do not cause severe environmental pollution. Moreover, they can be easily recovered after use through simple processes of pressure reduction or temperature decrease, thereby minimizing environmental impact. Supercritical fluid extraction technology not only enhances extraction efficiency and stabilizes carotenoids [117] but also reduces the environmental hazards associated with toxic reagents [118].

Table 2 shows the extraction of lycopene from grapefruit using supercritical carbon dioxide with rice bran oil as a co-solvent, as demonstrated by Dhakane-Lad et al. The extraction efficiency of lycopene was 70.52% [86]. Sun used rapeseed oil as a co-solvent, resulting in a two-fold increase in the extraction rate of β-carotene and a four-fold increase in lutein extraction compared to the supercritical carbon dioxide method alone [87]. The CO_2_-SFE approach at 40 °C and a constant CO_2_ flow rate (6 mL/minute) was used to extract the total carotenoids from *Rhodotorula* spp., red yeast, resulting in an average extraction rate of about 68.0 µg/g yeast (dry weight) [88]. Popescu et al. used camelina oil as a modifier to extract 203.59 mg/100 g of carotenoids from tomatoes using SFE at 450 bar and 70 °C [89].

Under the conditions of 318.15 K and 20 MPa, with a mass fraction of 5% ethanol, the number of carotenoids extracted from microalgae was 25 g/kg. This is a significant increase compared to the 6 g/kg extracted by supercritical carbon dioxide alone [90]. Additionally, the recovery rate of β-carotene in *Dunaliella salina* by supercritical carbon dioxide extraction was 90% under conditions of 500 bar, 70 °C, and 10 wt% ethanol as a co-solvent, resulting in reduced solvent costs [91]. Similarly, Xie et al. used ethanol-optimized CO_2_-SFE to extract about 421 μg/g of astaxanthin from flaxseed seeds under response surface optimization conditions at 41.6 MPa, 36.6 °C, and a 42.0% ethanol concentration [92]. However, Romano et al. found that reducing the concentration of ethanol to 10% avoided a significant decrease in the extraction number of carotenoids from tomato waste when combined with CO_2_-SFE [119]. These studies suggest that optimizing conditions for cooperative-assisted extraction using various methods is critical for extracting carotenoids.

### 3.4. Enzyme-Assisted Extraction

The EAE method utilizes enzyme-specific catalysis to selectively extract target molecules. Carotenoids are present in plant and yeast cells, with cell walls consisting mainly of cellulose, hemicellulose, and other structurally dense components. Mechanical methods can be used to break down the cell wall in combination with biological enzymes to enzymatically degrade the plant or yeast cell wall and extract carotenoids from the cell. The EAE technique employs mild reaction conditions and maximizes product activity [120]. Currently, the enzymatic extraction of carotenoids primarily involves the use of enzymes to break down cell walls, facilitating the release of carotenoids after the cell walls are ruptured. The enzymes currently employed for carotenoid extraction include pectinases, cellulases, and hemicellulases, which are cell wall-degrading enzymes [121]. Research by Homa et al. has revealed that the concentration of pectinase enzyme affects both the extraction of β-carotene and the antioxidant activity of the extracted β-carotene [122].

As shown in Table 2, the combination of pectinase and cellulase enzymes with ethyl lactate, due to ethyl lactate as a solvent, more easily penetrates moist raw materials, aiding in the enzymatic hydrolysis of plant cell walls. This increased the carotenoid content by 127 mg/kg (dry weight) and lycopene by 89.4 mg/kg (dry weight). This method is 6–10 times more efficient than conventional extraction methods [93]. Barzana et al. achieved a 97% carotenoid recovery under optimal conditions by recovering carotenoids from 80 L of pulverized *Tagetes erecta* (marigold) using simultaneous enzyme treatment and solvent extraction [94]. Lysozyme can also be used to extract carotenoids from *Rhodococcus* sp., although the extraction rate was only around 19% (25.52–33.40 μg/g) [95]. The ‘Stela’ tomato, commonly used in the canning industry, has waste tomato skins containing a high concentration of carotenoids. Using a mixture of cellulase (100 U/g) and endoxylanase (400 U/g) at 50 °C, researchers obtained total carotenoids (55.15 mg/100 g dry weight), β-carotene (35.85 mg/100 g dry weight), and lycopene (15.44 mg/100 g dry weight) [96]. The extraction efficiency of β-carotene from pumpkin was 61.75% when using a cellulase/pectinase ratio of 0.97 *w*/*w* at 42 °C for 92 min at pH 4.8 [123]. By combining EAE with a green solvent, carotenoid yield can be increased. In one study, a green organic solvent, 1:2 (*v*/*v*) menthol/lactic acid, was used to extract carotenoids from sunflower waste using a multi-enzyme complex under optimal conditions, yielding 1449 mg/100 g [98]. Dehydrated tomato skins were treated with rice bran oil and 1.4% Viscozyme L, which was a blend of β-glucanases, pectinases, hemicellulases, and xylanases, and incubated at 52 °C for 92 min, yielding a lycopene concentration of 399.6 mg/100 g [99].

### 3.5. Other Extraction Methods

Researchers are exploring novel extraction technologies such as PLE, which combines organic solvents to extract β-carotene, achieving a recovery rate of approximately 80% at high temperature and pressure for 20 min [100]. Experiments by Kwang et al. have shown that high-temperature PLE reduces the formation of harmful chlorophyll derivatives [124].

PEF, as an innovative technology, induces cell membrane electroporation, enabling the efficient extraction of intracellular antioxidant chemicals like carotenoids (see Table 2). Studies have shown that PEF intervention can increase the recovery rate of carotenoids to 85–90% [125]. In a study by Martínez et al., carotenoids were extracted from mucilaginous *R. glutinis* at 267 µg/g (dry weight) with an extraction efficiency of 80% using ethanol-assisted PEF [101]. Similarly, Georgiopoulou et al. extracted carotenoids from *Chlorella vulgaris* using MAE at 60 °C and 300 watts for 14 min. The additional amount of carotene (lycopene, β-carotene, and astaxanthin) was 7.06 mg/g, while the total carotenoids extracted amounted to 24.88 mg/g [102].

PEF technology has been employed for the extraction of natural compounds. Pre-treating raw materials with PEF can enhance the extraction efficiency of lycopene in subsequent solutions by 27–37% [126]. Ana et al. combined PEF with Accelerated Solvent Extraction to improve the recovery rate of astaxanthin [127]. As a sustainable extraction method, PEF not only increases extraction efficiency (39%) but also reduces the percentage of hexane in a hexane/ethanol mixture, thereby lessening environmental pressure [128].

Recent reports indicate that combining PEF and PLE technologies can increase the extraction rate and antioxidant capacity of antioxidant compounds, including carotenoids, thus enhancing their quality [129]. The integration of various extraction methods has become a trend for improving extraction efficiency.

## 4. Conclusions

The study of carotenoids, including lycopene, β-carotene, and astaxanthin, has led to an increased demand for high yield and purity in their extraction. This has necessitated improved extraction methods and the use of more environmentally friendly solvents.

The traditional extraction of natural compounds often requires large volumes of organic solvents, which can cause significant environmental damage. The excessive use of organic solvents also can reduce the quality and safety of the final carotenoid products, thereby limiting their potential applications. The adoption of green solvents as an alternative to traditional organic solvents can mitigate environmental impacts and enhance product safety. However, current green solvent systems are not sufficiently developed for widespread industrial adoption, with limited selectivity. Identifying an effective green solvent requires extensive experimentation to determine its compatibility, whether it should be used in combination and the optimal solute-to-solvent ratio. These factors currently constrain broader industrial implementation. Looking ahead, there is anticipation for the standardization of green solvent applications and the emergence of novel, faster, and more user-friendly alternatives.

Carotenoids are located within cells and require external stimuli to be released. The use of biological enzymes to break down cell walls offers high selectivity, gentle conditions, and ecological benefits. Physical technologies, such as ultrasound, microwaves, and a pulsed electric field, aid in the separation and extraction of carotenoids. These methods not only increase the extraction rate but also reduce the environmental impact of solvents. However, physical approaches require sophisticated equipment and occupy significant space, which are considerations that must be taken into account during production.

However, current methods for extracting carotenoids make it difficult to isolate specific types, such as lycopene, β-carotene, and astaxanthin. Biological methods for synthesizing carotenoids are expensive and have low yields. To increase the number of target products in organisms, further in-depth study is required on the structure and properties of these common carotenoids, as well as the main limiting factors in their biosynthesis processes. Developing extraction procedures that are convenient, fast, environmentally friendly, and efficient is critical.

## Figures and Tables

**Table 1:** Molecular structure and biological activity of carotenoids.

Carotenoids	Structure	Physiological Function	References
Lycopene	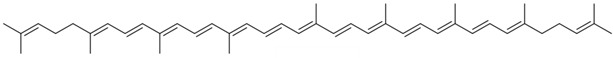	Serum lipid reduction	[24]
		Preventing reproductive toxicity	[25]
		Preventing prostate cancer	[26]
		Cardiovascular disease prevention	[27]
		Neuroprotection	[28]
		Heightening insulin levels	[29]
		Inhibiting gastric cancer	[30]
		Elevating muscle attributes	[31]
β-carotene	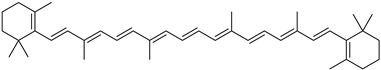	Decreasing signs of type-2 diabetes	[32]
		Boosting the immune system	[33]
		Preventing macular degeneration	[34]
Astaxanthin	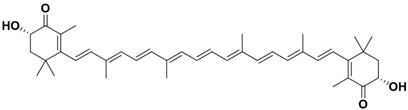	Suppressing pro-inflammatory cytokines	[35]
		Increasing UV resistance	[36]
		scavenging superoxide anions	[37]
		Promoting collagen production	[38]
		Enhancing fertility and hatchability	[39]
Lutein	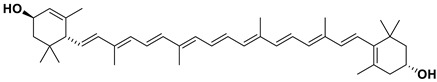	Enhancing shrimp growth	[40]
		Promoting development	[41]
		Protecting lipids	[42]
		Protecting the neurological system	[43]
Canthaxanthin	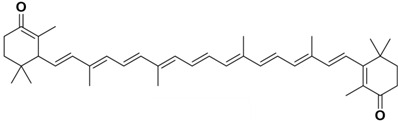	Counteracting oxidative stress	[44]
		Substituting astaxanthin	[45]
		Improving chicken fertility	[46]
Zeaxanthin	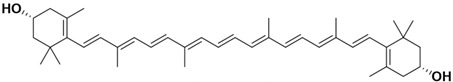	Increasing catalase and insulin levels	[47]
		Counteracting light and oxidative side effects	[48]
		Slowing cataract progression	[49]
		Enhancing oxidative capacity	[50]
		Regulating the gut microbiome	[51]

**Table 2:** Extraction methods of several typical carotenoids.

Extraction Method	Source	Kingdom	Extracted Substance	Solvent	Whether Green Solvent	Extraction Efficiency	References
Solvent extraction	Tomato waste	Plantae	Lycopene	N-hexane/acetone 1:3	No	98.3%	[19]
	Pericarp of wood betel seed	Plantae	Carotenoids	Ethyl acetate	No	271 mg/100 g (dry weight)/150 min	[73]
	*Citrus sinensis* peels	Plantae	Carotenoids	Eucalyptus oil/menthol 1:1	Yes	359.3 ± 3.5 mg/100 g (fresh weight)/20 min	[74]
	exoskeleton	Animalia	Astaxanthin	Hexane/acetone 1:1	No	8.5 mg/g	[75]
	*Rhodosporidium toruloides*	Fungi	Carotenoids	Alcoholic/hexane	No	78.7%	[76]
UAE	Tomato waste residue	Plantae	β-carotene	FAEE	Yes	49.7 mg/100 g/15 min	[77]
	Tomato waste residue	Plantae	Lycopene	FAEE	Yes	101.4 mg/100 g/6 min	[77]
	Papaya	Plantae	Carotenoids	Vegetable oils and SUPRAS	Yes	1 mg/g (dry weight)/5 min	[78]
	Tomato waste	Plantae	Carotenoids	Hexane/ethanol	No	yield Increased 143%	[79]
	Pomegranate waste	Plantae	Carotenoids	Vegetable oils	Yes	93.8% (0.3255 mg carotenoids/100 g of dry peels)	[80]
	*Chlorella vulgaris* and *Purpuricus porphyrinaceus*	Plantae	Carotenoids	Ethanol/eexane 2:1	No	6435.60 µg/g	[81]
	Orange peels	Plantae	Carotenoids	Menthol/camphor	Yes	653.5 mg/100 g (fresh weight)/20 min	[82]
	Carrot pomace	Plantae	Carotenoids	51% ethanol	Yes	31.82µg/g/15 min	[83]
	*Rhodotorula glutinis*	Fungi	Carotenoids	Aqueous medium	Yes	25 mg/L	[84]
UAE + MAE	Seabuckthorn pomace	Plantae	Carotenoids	Edible oils	Yes	26.91–34.35 mg/100 g	[85]
CO_2_-SFE	Pink grapefruit	Plantae	Lycopene	Rice bran oil	Yes	70.52%	[86]
	Carrots	Plantae	β-carotene	Rapeseed oil	Yes	increased by 2 times	[87]
	Carrots	Plantae	Lutein	Rapeseed oil	Yes	increased by 4 times	[87]
	*Rhodotorula* spp.	Fungi	Total carotenoids	Solvent-free	/	68.0 ± 1.4 µg/g yeast (dry weight)	[88]
	Tomato	Plantae	Carotenoid	Camelina	Yes	203.59 mg/100 g	[89]
	*Dunaliella salina*	Plantae	Carotenoids	5% ethanol	No	25 g/kg	[90]
	*Dunaliella salina*	Plantae	β-carotene	10 wt% ethanol	No	90%	[91]
	Camelina seeds	Plantae	Astaxanthin	42.0% ethanol	No	421 ± 14 μg/g	[92]
EAE	Tomato waste	Plantae	Carotenoids	Pectinase and cellulase enzymes	/	raised by 127 mg/kg (dry weight)	[93]
	Tomato waste	Plantae	Lycopene	Pectinase and cellulase enzymes	/	were raised by 89.4 mg/kg (dry weight)	[93]
	Marigold	Plantae	Carotenoids	Macerating enzymes	/	97%	[94]
	*Rhodococcus* sp.	Fungi	Carotenoids	Lysozyme	/	25.52–33.40 μg/g	[95]
	Bulgarian tomato peels	Plantae	β-carotene	cellulase (100 U/g) and endoxylanase (400 U/g)	/	35.85 mg/100 g (dry weight),	[96]
	Bulgarian tomato peels	Plantae	Lycopene	Cellulase (100 U/g) and endoxylanase (400 U/g)	/	15.44 mg/100 g (dry weight)	[96]
	Pumpkin	Plantae	β-carotene	Cellulose/pectinase ratio is 0.97 *w*/*w*	/	61.75%/92 min	[97]
	Sunflower waste	Plantae	Carotenoids	Multi-enzyme + menthol/actic acid 1:2 (*v*/*v*)	Yes	1449 mg/100 g	[98]
	Tomato peels	Plantae	Lycopene	1.4% Viscozyme L + rice bran oil	Yes	399.6 mg/100 g/92 min	[99]
PLE	Carrot	Plantae	β-carotene	Ethanol	No	80%	[100]
PEF	*Rhodotorula glutinis*	Fungi	Carotenoids	Ethanol	No	213.6µg/g (dry weight)	[101]
MAE	*Chlorella vulgaris*	Plantae	Total carotenoids	90% ethanol	No	24.88 mg/g	[102]

## Data Availability

No new data were created or analyzed in this study. Data sharing is not applicable to this article.

## Abbreviation

UV, ultraviolet; GPP, geranyl diphosphate; GGPP, geranylgeranyl diphosphate; MVA, the mevalonate pathway; MEP, the 2-C-methyl-d-erythritol-4-phosphate pathway; MAE, microwave-assisted extraction; UAE, ultrasound-assisted extraction; SFE, super-critical fluid extraction; PLE, pressurized liquid-assisted extraction; PEF, pulsed electric field-assisted extraction; EAE, enzyme assisted extraction; DES, deep eutectic solvent. FAEE, fatty acid ethyl esters; and SUPRAS, supramolecular solvents.
